# Using performance-based regulation to reduce childhood obesity

**DOI:** 10.1186/1743-8462-5-26

**Published:** 2008-11-18

**Authors:** Stephen D Sugarman, Nirit Sandman

**Affiliations:** 1 School of Law, University of California, Berkeley, USA

## Abstract

**Background:**

Worldwide, the public health community has recognized the growing problem of childhood obesity. But, unlike tobacco control policy, there is little evidence about what public policies would work to substantially reduce childhood obesity. Public health leaders currently tend to support traditional "command and control" schemes that order private enterprises and governments to stop or start doing specific things that, is it hoped, will yield lower childhood obesity rates. These include measures such as 1) taking sweetened beverages out of schools, 2) posting calorie counts on fast-food menu boards, 3) labeling foods with a "red light" if they contain high levels of fat or sugar, 4) limiting the density of fast food restaurants in any neighborhood, 5) requiring chain restaurants to offer "healthy" alternatives, and 6) eliminating junk food ads on television shows aimed at children. Some advocates propose other regulatory interventions such as 1) influencing the relative prices of healthy and unhealthy foods through taxes and/or subsidies and 2) suing private industry for money damages as a way of blaming childhood obesity on certain practices of the food industry (such as its marketing, product composition, or portion size decisions). The food industry generally seeks to deflect blame for childhood obesity onto others, such as parents and schools.

## Proposal

We propose a radically different approach we call "performance-based regulation". This policy strategy rests on the moral argument that the food industry must take responsibility for child obesity consequences that flow from the consumption of the products from which they profit. It is a regulatory intervention that seeks to harness private initiative in pursuit of the public good. But it resists telling industry specifically what to do to address the problem. Instead, food companies are to be assigned outcome improvements in the form of reduced childhood obesity. Failure to achieve the regulatory target would result in substantial financial penalties. The idea is to align the economic interests of food companies with sharply lower childhood obesity rates. Administratively, food and beverage companies would be assigned a share of the problem based on the overall share of the "bad calories" they sell. In order to make administrative oversight practical, the regulated firms would be assigned specific geographically clustered schools at which they will be required to lower the obesity rate. Firms would be given a reasonable period of time to phase in their childhood obesity-reducing strategies.

## The problem of childhood obesity

Childhood obesity is a large and growing problem in many countries. In New South Wales, Australia, for example, the obesity rate among schoolchildren has risen to about 25% (one in four children), up from 11% two decades earlier [1-3]. Similarly, in the United States, the obesity rate among schoolchildren is three times what it was 35 years ago [4]. Sadly, while hunger remains a grave problem for children in many places around the world, in wealthier nations the opposite phenomenon is taking center stage.

Both Australian and US studies have found childhood obesity to be an important public health problem because it leads to high rates of type 2 diabetes in children, as well as high blood pressure – an early indicator of cardiovascular risk [5-7]. In 2007, the Australian Government prioritized obesity prevention as a National Health Priority Area, ranking it alongside many of the diseases it contributes to, including cancer, cardiovascular disease and diabetes [2].

Currently, health promotion and prevention campaigns are the preferred policy approach in Australia. This includes regulations of television advertisements of unhealthy foods/eating habits directed at children [7], promoting leisure time sports and other activities for youth [8], and food labeling programs such as the National Heart Foundation's healthy "tick" program. [7]. Some argue, however, that major legislative and other regulatory measures are required [8,9]. But exactly which public policy interventions have the best chance of sharply lowering the proportion of children who become obese? In the abstract, we know that obesity results from too much caloric intake and too little exercise. But how can society best help children keep these two factors in better balance? At the moment, nobody really knows.

This is in marked contrast to the arena of tobacco control – another serious public health problem throughout the world. Studies show that policy interventions can make a difference in the smoking rate through a combination of actions, the most important of which appear to be 1) substantial cigarette taxes 2) tough regulation of the physical spaces where smoking is permitted, and 3) aggressive publicly sponsored counter-advertisements highlighting the public fraud perpetrated by tobacco company executives regarding the dangers of cigarettes and their addictive quality [10]. Perhaps over time we will learn what works best to contain childhood obesity as well, but we are not there yet. It seems likely that the best public health approach to obesity is prevention. After all, the very existence of a huge "diet industry" aimed at adults demonstrates that losing weight may be at least as difficult as quitting smoking. Put differently, an important parallel between obesity and tobacco addiction is that, with both, once afflicted, it is difficult to avert the danger, and that, just as most smokers begin smoking as teenagers, a large number of obese adults were already obese as children.

To be sure, it is not that people have no idea as to what to do about childhood obesity [11]. And it is not that various countries and localities are not trying out different strategies [9]. Rather, the problem is that we (currently) lack evidence as to what exactly will sharply reverse the alarming trend of recent years.

## A new idea: performance-based regulation

We propose an approach we call performance-based regulation. It is a regulatory intervention that seeks to harness private initiative in pursuit of the public good. Briefly stated, a regulatory agency assigns companies performance targets in the form of reduced harm to the public health. The companies are not to be told specifically what to do to address the problem, but can use whatever methods they found most effective and efficient. Failure to achieve the regulatory target would result in substantial financial penalties.

Performance-based regulation is increasingly becoming the favored strategy for addressing other problems, including, for example, certain pollution problems. On a smaller regional scale, power plants are instructed that only so much pollution may be emitted from their stacks, with the amount lowered each year. It is left up to the firms to figure out how best to achieve the regulatory outcome goals [12]. On a much larger scale, with respect to global warming, the growing consensus seems to be that outcome goals should be imposed on nations, who must then find the right solution for meeting their goals [13-15]. In turn, countries are looking primarily to the business sector to implement the most efficient mechanisms to reduce society's overall carbon footprint.

Another example in the United States is the shifting of elementary and secondary schools to a performance-based regulatory scheme. Under the No Child Left Behind Act, schools are held accountable for achieving specified educational outcomes for their pupils, with legislators specifying fewer input requirements (like class size, teacher quality measures, etc.). Instead, under this law, it is up to the schools to figure out how to solve the problem for which they have been given responsibility [16]. Before explaining how performance-based regulation could work with respect to childhood obesity, we first discuss the rationale underlying the approach, the possibility of its political acceptance, and alternatives to it.

## Framing childhood obesity as the responsibility of the food industry

Underlying performance-based regulation is the moral argument that product-makers are, in large part, rightfully responsible for the negative health consequences of the products they sell. In the case of childhood obesity, the food industry must take responsibility for the child obesity consequences that flow from the consumption of the unhealthy products from which they profit.

As awareness of childhood obesity spreads, various interests fight over how this problem should be "framed" [9,11]. The strategy of the food and beverage companies is to frame this as a problem of personal choice and parental failure [17]. Their narrative goes something like this: families are not taking responsibility for the health of their children, and they should so do. Parents should make sure their children exercise enough and do not eat too much. No individual food or type of food should be blamed, since it is all a matter of moderation. If parents do not understand this, then it is the responsibility of doctors and other health professionals to make it clear to them. As for teenagers, schools should educate them as to the need to eat and exercise properly. Almost any (proposed) regulation beyond that is quickly equated with the "nanny" state, in which public health fanatics step in to usurp (or worse, to undermine) family responsibility, while at the same time infringing on the freedom of responsible adults [18,19].

By contrast, we believe public health strategists must frame childhood obesity as centrally the responsibility of Big Food, just as childhood smoking has been effectively framed in many countries as the responsibility of Big Tobacco. In the past, relying largely on parents to control their children's smoking resulted in unacceptably high teen smoking rates. More recently, public health activists have blamed tobacco companies for marketing cigarettes to children and have cast teen smokers (and their parents) as victims. In this light, they paved the way for government to intervene to change social norms about smoking [20,21]. To this end, a range of measures were pursued under the banner of holding Big Tobacco responsible.

But what precisely would it mean to frame childhood obesity as the responsibility of Big Food? Our proposal calls for performance-based regulation. This approach orders the food and beverage industry to tackle and contribute significantly to the solution of the problem its products have created. What our plan demands of these firms is results – reduced childhood obesity rates – leaving it to them to figure out how best to achieve the socially desired public health gains.

## Politics

Performance-based regulation is likely to be opposed initially by both public health traditionalists and private enterprise. The former generally distrust business and tend to favor a "professionals-know-best" approach to regulation. The latter fear lost profits and, as just noted, are eager to frame childhood obesity as somebody else's problem. Yet, in the hands of the right entrepreneurial politicians, performance-based regulation could become a compromise solution that triumphs over the alternatives.

On one end of the political spectrum are those conservative leaders who follow the line of the food industry and are ideologically inclined to treat childhood obesity as a matter of personal responsibility of individual families. But the reality in many countries is that obesity threatens a gigantic health care cost burden that will fall on the entire society. The same limited government intervention with respect to childhood obesity of the sort that has taken place over the past two decades will simply not suffice. The parallel here to greenhouse gas emissions and climate change is striking. Even the most reactionary national leaders are beginning to acknowledge that some sort of collective action to fight global warming is required.

On the other end of the political spectrum, political leaders who are quite willing to back an activist approach to public health problems confront two important challenges already noted with respect to childhood obesity. First, there are no evidence-based policy interventions for them to support that are reliably going to reduce childhood obesity rates in a significant way. Second, sensible experimental interventions risk being immediately labeled by opponents as "nanny" state strategies that interfere with personal liberty.

Performance-based regulation can bridge this divide. Government acts, but it does not tell business or consumers what to do. Business is enlisted in support of the public good and so can be cast in a positive light. The market is relied upon, but society sets the goals for the market to maximize. As already noted, this is just how leaders in many parts of the world are now trying to confront climate change, and, as with global warming, if business leaders realize that the political alternative is likely to be government agencies telling them how to run their companies, performance-based regulation can become far more attractive.

Public health leaders may also come to appreciate that, even though performance-based regulation may undercut the political power of a few elite professionals, it should nonetheless open up many opportunities for local public health experts to consult with business on how to achieve their childhood obesity targets.

Of course, the food industry will object to being the object of regulation on the ground that food is hardly the only cause of childhood obesity. Yet, one of the beauties of performance-based regulation is that the food industry would not be asked to eliminate the problem, but instead only significantly to reduce it. Here again, political framing is key. To win public support for performance-based regulation, its supporters will probably first have to convince opinion leaders that, because junk food companies are profiting financially from selling products that harm the health of our children, it is only fair to start by asking those companies to do their share in reducing childhood obesity.

## Alternatives

Before explaining our scheme in detail, we want to contrast it with other approaches that rely on a similar framing which holds food companies responsible for childhood obesity. First, people may hold food and beverage companies responsible by successfully suing them in product liability lawsuits based on tort law, thereby obtaining money damages for obese children (a similar approach has been tried, though not very satisfactorily in our view, with respect to cigarettes [22-24]). The theory is that tort litigation will expose misconduct by the food and beverage industry (such as misrepresenting products as healthy when they are not) and that tort law's deterrent power will alter the behavior of food companies, thereby lowering the childhood obesity rate.

However, there are serious drawbacks to this approach. First, at the individual tort claim level, it will be nearly impossible for obese children to lay responsibility at the feet of any specific food or beverage company. After all, almost everybody eats and drinks a variety of products made by many different firms. Second, even if some showing of wrongdoing could be proved, it is by no means clear that eliminating the behaviors that the legal system would be willing to label as "faulty" would make significant headway on the childhood obesity problem. Instead, tort law's doctrine of strict liability would have to be radically expanded to hold food and beverage companies liable for the consequences of their products regardless of fault. Yet, common law courts in both Australia and the United States have been unwilling to embrace this position even as to tobacco [22].

A second very different approach to holding food and beverage companies responsible is to adopt specific regulatory measures for curtailing irresponsible firm behavior, perhaps also insisting on new behaviors that the regulators believe should be engaged in by responsible companies. In a sense, this approach uses the legislature or an administrative agency to pick out acts of commission or omission that the judicial system might hold worthy of tort liability. This traditional regulatory approach, termed "command and control", has some advantages over tort law. First, it does not require proving an individual causal link to a specific child's obesity. Second, the prohibited and required acts need not be cast as narrowly as would be necessary to satisfy the conventional standards of negligence law. Yet, as already noted, the problem with this approach is that regulators do not actually know what changes to industry behavior would make significant advances toward achieving the ultimate social goal – lower childhood obesity rates.

A third approach to childhood obesity takes its cue from tobacco control, by trying to change behaviors through price effects [25]. This entails making junk food that children eat much more expensive while sharply reducing the price of the healthier food they should be eating. A straightforward approach would be to levy high taxes on junk foods generally and use the proceeds to subsidize the supply of nutritious food. Imposing the tax could be framed as a way of holding junk food sellers responsible.

This approach could be tailored to target products heavily consumed by children, such as sugared breakfast cereals and the candy and chips consumed by teens during or just after school hours. Since childhood obesity is, alas, disproportionately a problem for children from lower income families in both Australia and the United States [6,11], another price-change strategy might be to offer target subsidies of healthier food to the poor. In the United States, the "food stamps" program currently provides low-income households with electronic debit cards to use for the purchase of eat-at-home foods. Hence, one policy idea is that when the card is used to buy healthy foods, the cardholder should be charged only a share of the cost, whereas when unhealthy food is bought, the cardholder could be charged extra [27].

Notice, however, that, as with the command and control proposals described above, this price-influencing strategy is aimed essentially at inputs, not outcomes. Nothing about the policy interventions actually requires that fewer children wind up obese. Rather, it is simply assumed or hoped that these sorts of strategies will result in obesity prevention.

Our proposed strategy, performance-based regulation, is quite different. It insists on results. It does not depend on "experts" or government actors to know or decide what are the most promising changes to order. It leaves that up to the regulated parties to decide. What it asks of them is success in the form of changed outcomes – in this case, obesity prevention as measured by reduced childhood obesity rates. Rather than tort law, rather than tax and subsidy schemes, and rather than specific input-oriented command and control requirements, the regulatory scheme would instead demand outcomes.

## Performance-based regulation applied to childhood obesity

We propose a regime of performance-based regulation of sellers of non-nutritious food [28]. As already noted, because junk food consumption is one central cause of childhood obesity, we think the most promising first step is to persuade opinion leaders that food sellers may be fairly asked to take responsibility for the consequences of their products. Also, we focus on children for two reasons. First, it is difficult to argue that they, as compared with adults, are making mature judgments about what and how much to eat [29]. Second, because we believe that prevention is far more promising than weight loss, it makes sense to start when people are young and before they are overweight.

Some have suggested that the parties being regulated need not be the food and beverage companies. Why not parents? Why not schools? Or states? We find those variations less attractive. For one thing, this undermines the original framing goal of casting childhood obesity as the responsibility of the food companies that sell junk food. For another, direct financial penalties of schools or parents who do not meet their goals would be offensive in ways that penalties imposed on businesses might not be. (Indeed, unlike parents or public schools, businesses would be able to treat such penalties like a tax that could be passed on in the price of the bad food they sell.)

As already noted, we do not insist that food-sellers eliminate childhood obesity. This concession serves both to acknowledge that such a goal would be too ambitious and that other factors contribute to childhood obesity. Instead, we recommend a regulatory goal of a 50% reduction over 10 years. Were that achieved, it would be an enormous public health gain and would return nations to the childhood obesity rates they had decades ago.

### (a) Determining which food companies are covered

We would allow a year from the time the plan is adopted until it goes into effect. One reason for this delay is that it would take the regulatory agency in charge some time to put the scheme into place. First, the agency would have to determine which food-sellers are covered. We do not propose imposing this form of regulation on all food-sellers. After all, some food is healthy and nutritious. Instead we direct the regulatory scheme at sellers of "bad food", defining bad food as that which derives either 40% of its calories from sugar or 30% of its calories from fat. These definitions are roughly equivalent to those used by other health advocates in defining junk food.

Another purpose of the delay is to permit some bad food-makers to reformulate their product so as to remove it from the regulated category. Some might be concerned that bright-line thresholds give firms an incentive to reformulate products so that they just barely avoid regulation, say, by lowering the fat level to 29% or the sugar to 39%. Yet, widespread action of this sort alone could lead to important public health gains. Moreover, even if the regulator does not consider this a substantial enough gain, it could choose to start with lower thresholds.

Our plan would exempt smaller firms because they would be too numerous to keep a regulatory eye on. However, many food products sold by small firms would nonetheless be included in the regime. This is because of the way responsibility for bad food is assigned. A manufacturer of bad food carrying the manufacturer's name (like Coca-Cola) would have responsibility for that product, whether sold through retailers, in restaurants, or the like. Large retailers (like supermarkets – Coles and Woolworths in Australia – and fast food chains like McDonald's everywhere) would be responsible for all the bad food they sell that is not associated with large brand-name manufacturers. Hence, the only bad food that would escape assignment to a participating firm would be that made by small firms and sold through small firms. Were this thought a serious problem, it might be addressed by assigning the responsibility for such foods to the large distributors who serve as middlemen in the food chain.

### (b) Determining how much of the problem each company responsible for

The next job is to determine the market share of the covered bad food held by each covered seller. For these purposes we would include all bad food unless the seller can demonstrate that it is not marketed to children and that its consumption by children is de minimus. Moreover, we would then presume, for ease of administration, that children eat proportionately the same share of each covered food as do adults. Were this presumption thought to be significantly unfair to a substantial number of food companies, more administrative effort could be put into determining the bad food market with respect to children and the shares held by each covered seller.

The overall bad food market probably should not be defined by price or sheer volume, however. Rather, we suggest defining it in terms of the total amount of bad calories. For example, if 80% of the calories in a bottle of Pepsi come from sugar, then the calories it contains beyond the 40% cut-off for inclusion in the plan would be deemed bad calories. In this way, the more a product exceeds the nutritional baseline for regulation, the greater share of the bad calorie market is assigned to it. Once the total amount of bad calories contributed by all covered products is determined, a proportionate share of that total could be assigned to each product. Then, the various products sold by each participating seller can be aggregated, with the result that each regulated firm would be assigned its share of the overall market in bad food.

Hypothetically – and merely for purposes of illustration – suppose that major sellers wind up with these sorts of shares of overall responsibility: Coca-Cola 10%, Pepsico 10%, Unilever 5%, McDonald's 5%, Nestle 5%, Cadbury 5%, and so on. It seems clear to us, however, that if Coca-Cola were, for example, deemed to be responsible for 10% of the childhood obesity problem it cannot be directed to solve 10% of the problem for every child in the nation because the regulatory agency could never tell whether or not Coca-Cola had achieved its goal. This is in contrast to the possible use of performance-based regulation, say, to reduce highway fatality rates. There, if Toyota were told, for example, to reduce by 25% the number of people killed on the roads while in Toyota-made vehicles, the relevant agency could readily measure whether or not Toyota had achieved its regulatory target.

Therefore, when it comes to childhood obesity, we have had to develop a different strategy. Instead of asking Coca-Cola to take partial responsibility for keeping slim all the targeted children, we think it would be much more sensible to assign specific pools of children to Coca-Cola for whom that enterprise would be 100% responsible. In this way we would measure Coca-Cola's success in terms of obesity rate changes among their assigned pool of children (who would make up 10% of the relevant population assuming Coca-Cola is deemed responsible for 10% of the problem).

We propose doing this by assigning each regulated firm a set of geographically clustered schools, and, in turn, the pupils who attend those schools. For example, in Australia, Coca-Cola might be assigned all the schools in Queensland, or in the United States, all the schools in four or five states in the southeast where it has its national headquarters.

After further consideration, we decided it would be best not to include all schools in the scheme. Instead we propose to focus the plan only on schools with existing obesity rates that are higher than the end-of-plan target obesity rate. For example, assume that a nation's current childhood obesity rate is 16%, and that the goal for schoolchildren generally is 8% at the end of 10 years. On this assumption, we suggest that only schools that already have obesity rates above 8% would be included. Generally speaking, these schools would tend to have disproportionately more children from lower income families, and that would protect the plan from the risk that firms might otherwise elect to concentrate their efforts primarily on children from more affluent families.

### (c) Measuring success

Notice that under our proposal the regime would not track specific children. Indeed, children who are in the second year of school or higher at the time the plan first goes into effect would probably be out of school by the end of the regulatory period and would not be counted then. Rather, at the start of the scheme, most of the children who will be targeted by participating firms would be either six years old or younger or not yet born. From a prevention strategy, then, the regulated firms would have a clear incentive to focus not only on primary school children, but perhaps more importantly on pre-schoolers, and to keep a large share of them from becoming obese in the first place. To be sure, as those children age, firms would have to make an effort to maintain those children's non-obese status. Hence, over time, a firm would follow the children for whom it is responsible as they pass from primary schools to their geographically related secondary schools.

While other ways of defining "success" are clearly possible, we recommend focusing on a simple yes/no criterion that would use an agreed upon body-mass-index (BMI), the current standard measure of obesity in the profession and the basis on which the prevalence rates quoted earlier are calculated. This means that a firm would get no credit for a child who is just slightly obese and would get full credit for one who is just barely non-obese. Probably, however, a large share of the target children would be well on either side of the line, and to be safe, participating firms would have an incentive to have as many of its children as possible well below the cut-off BMI.

Under our proposal, the scheme would not wait 10 years to see whether participating firms had achieved their goals. On the other hand, we think that it would be inappropriate to insist on immediate results, as it might take the regulated firms a while to organize their campaign, begin to learn what interventions show promise, and so on. Hence, we propose that no penalties apply until the end of the fourth year of the plan.

### (d) Later modifications to the scheme

For now we leave open the question of what should be done after the end of the tenth year. If the plan were a success, it might be quite desirable to carry it forward. If nothing else, that would imply a re-determination of the bad food market and individual firms' market shares, as those probably would have shifted somewhat over time – as new products enter the market, as some firms restructure their products to be outside the bad food definition, and as consumption patterns change. Also, some previously exempt firms might grow large enough to be covered in a subsequent cycle. Furthermore, policy-makers by then might decide to re-define bad food. Perhaps a lower threshold of fat and sugar would be used. Perhaps a more complex definition would be adopted. Perhaps other sorts of firms could also be included in the regulatory process; for example, firms whose products contribute to the excessively sedentary lifestyle of many children.

In addition, experience gained during the first ten years would help determine the appropriate targets for a second cycle. Might it be reasonable to ask firms to reduce childhood obesity rates even further, perhaps down to 5% from 8%? Would it be thought sufficient merely to insist that an 8% rate be maintained (assuming the goal of the first cycle were achieved)? Also, perhaps a different set of schools would be included in a subsequent cycle.

Moreover, we acknowledge that it might turn out that performance-based regulation was not very successful in achieving the obesity reduction goals we hoped for, in which case it might well be wise to attempt a different regulatory scheme. However, if government were to try our scheme, it should do so for a full initial cycle and commit at the outset, as firmly as possible, to sticking with the plan for the full ten years. Otherwise, the risk is that firms will not take their responsibilities seriously and will instead focus their political energy on overturning the plan.

To be sure, by the end of the first ten years we will have a lot more information about which intervention strategies seem successful at preventing childhood obesity and which do not seem to work very well. This will tempt some of those with governmental power to simply order the adoption of those successful interventions after year ten (or even earlier), rather than to engage in a new round of performance-based regulation. We are not sure this would be wise, however.

One of the key assumptions underlying our strategy is that regulated firms will experiment with different approaches, will begin to learn which work better, will learn from each other, and will thus move towards efficient and effective means for dealing with the childhood obesity problem. If this approach works well, that might be a very good reason for continuing with it. After all, even if techniques A and B seemed very successful in the first ten years, maybe firms will learn that methods C and D are even better, or better for dealing with still lower obesity targets that may be imposed later on. The problem with command and control schemes is that, in prescribing particular measures, we risk that regulators will impose outdated technology.

### (e) Setting a penalty

What if firms fail to meet their obesity-reduction targets? For performance-based regulation to have teeth, enterprises falling short of their obligations must be penalized. This will be a monetary fine, which, in effect, becomes a tax. As noted already, we would begin penalties after the fourth year of firm participation and continue penalties through the end of the first ten-year cycle. In our proposal, firms would continue to have the target number of obese children in the schools in their jurisdiction reduced each year. This means that, if they fall short of their goal at any point, in the following year they will have to make up the shortfall in addition to achieving the next year's required gain to get back on schedule.

The amount of the penalty should be set at a level which makes it financially more attractive for a firm to invest in obesity prevention than simply to pay the fine for non-compliance. Of course, it is not socially desirable to prod firms to spend excessively on obesity prevention, although there would surely be debate over what is excessive. In theory, spending should be encouraged up to the level of the social benefit achieved by prevention. Assuming increasing marginal costs of obesity prevention, if the chosen per-child penalty equaled the per-child average social cost and the target reduction were set at what could be cost-effectively achieved, a rational participating firm would be enticed to invest up to the efficient level of prevention, would achieve that outcome, and would not pay a penalty.

In practice, however, it will not be possible to determine in advance the precise social cost and prevention cost. Therefore, both the efficient penalty and the efficient level of investment it induces are also unknown. In turn, the obesity reduction target we propose may be too meager or too ambitious. In response to this problem, our view is that, where necessary, the plan should overreach with respect to both the target and the penalty. If the target is set too high, we may be able to learn how much is reasonably possible, especially if the penalty is also too high. This, in effect, stimulates the regulated firms to explore and reveal a larger portion of their cost curve. Otherwise, if the target is too low we risk having them simply stop when they reach the target even if it would be socially desirable and economically efficient to reduce obesity rates further. So too, a low penalty might in fact be lower than the social cost, inducing firms to quit before reaching the efficient level of prevention.

To be sure, if the penalty is too high and the target level too ambitious, firms might inefficiently over-invest in prevention; but how bad would this be? And if the costs of prevention at some point exceed the penalty amount, the upshot would be that were a firm still below its target, it would from there on simply pay the penalty. At that point our plan would be converted into one that imposes a tax on bad food, which ought to be socially attractive in its own right on the ground that social costs of bad food would be internalized into its price.

### (f) Freedom to choose how to reach target

As emphasized several times already, performance-based regulation insists on results, but leaves it up to the regulated parties to decide what obesity-reducing strategies to employ. If school officials would be effective in enticing children to remain non-obese, under our plan the regulated firms could team up with the schools for which they are responsible to take advantage of that approach. Or, if it were more effective to reach out to children and families themselves outside the school setting, or to attack the problem via television or changes in the children's environment, firms would have the flexibility to pursue this method. These firms, after all, unlike governmental bodies or families, are in the business of pursuing results while keeping costs down.

It is critical to appreciate that under our proposal a firm like Coca-Cola has no special incentive to reduce consumption of its products nationwide or even to reduce the sugar in its products. First, Coca-Cola would not get any credit for any social benefit that might occur outside its geographic area of responsibility. Also, Coca-Cola may well want to pursue the idea that children under its responsibility can be non-obese and still consume its products in amounts they do today. And, so far as our plan is concerned, that outcome would be fine. On the other hand, it might be that Coca-Cola and its major sweetened beverage competitors who are regulated by the plan would want to get together to agree on some national strategy that would benefit each of them in the geographic area where they are regulated. To the extent permitted by antitrust laws, all of the firms might agree, for example, on reductions in the size of sweetened beverage portions sold and served to children.

Moreover, to the extent that the regulated firms believe public policy changes are necessary, they can jointly lobby for those reforms; thus, the political efforts of the regulated firms would be aligned with public health goals instead of being focused on profits for the enterprise, as they are now. This means there is reason to expect that the regulated firms would more willingly cooperate with local public health officials than they do now. At present, business efforts said to be advancing public health are all too often disguised marketing campaigns or "voluntary" strategies aimed at precluding coercive regulation. But if performance-based regulation were put in place, businesses would need to achieve actual public health gains and, to the extent that local public health experts made good partners, they should be eagerly sought out.

A potential pitfall of performance-based regulation is that it can entice firms to act in ways that are not socially desirable. Therefore, the agency in charge of administering the program would have to be empowered to ban certain practices and to penalize firms that acted in socially perverse ways. One concern with all performance-based strategies is that the objective that is being measured is not actually the precise social goal. This raises the question of whether a certain BMI level is actually what is desired or whether some other definition of obesity or poor health would be a better goal; were the latter true, firms would be striving for the wrong outcome. A second concern is that firms might seek to push some obese children out of the schools in their target pool. A third concern is that firms might press children in their pool to engage in dangerous activities in order to keep from becoming obese. After all, we do not want to promote eating disorders through this scheme.

For this reason, we recommend that firms be required to disclose to the regulatory agency the nature of their plans to deal with the obesity rates of the children in their pool. This would allow the agency to veto socially unacceptable strategies. While this is a different role from that which many public health agencies traditionally play, we think it is a role for which regulatory agencies, when properly staffed, are well suited. After all, government has lots of experience with somewhat analogous problems, such as the regulation of child abuse and neglect by those (parents and organizations) having custody of children. In any event, the risk that firms would embrace socially perverse strategies should not be exaggerated. Note well that the lengthy duration of the regulatory cycle provides an incentive for firms to introduce children to sustainable weight-control strategies that would stave off obesity for years, not months.

## Conclusion

Compared with traditional alternatives, performance-based regulation is a promising new approach to tackling the growing problem of childhood obesity. Although we have designed and described our plan as though it would be adopted as a national scheme covering, say, all of Australia, in populous enough countries we could imagine it being adopted regionally on an experimental basis. For example, in the United States, California alone might go for the performance-based regulation strategy.

While our proposal will cost public money to administer, it is likely that not all firms will reach their obesity-reduction targets and will pay penalties. Those payments can first be used to support activities of the regulatory agency. On the other hand, if our plan is so successful as to result in but a few or even zero penalties being imposed, then it will have been an enormous public health success. In that event, surely it would be worth paying for the administrative costs of the scheme out of general revenues.

Performance-based regulation also presents regulated firms with certain advantages. For example, we believe that from the perspective of corporate image, a firm would much prefer to have the bragging rights that come from reducing childhood obesity rates in the area of its responsibility than the shame that often comes from being successfully sued in tort. So, too, we think that firms would prefer the flexibility of preventing childhood obesity in ways they think best rather than taking specific orders from government regulators.

Our goal here has been to present the case for performance-based regulation and its potential application to the public health problem of childhood obesity. We have tried to acknowledge some potential problems with our approach, while emphasizing our belief that they are outweighed by its advantages (as well as offering our view that many potential problems can be avoided or minimized by careful drafting of the plan's details). In the end, perhaps the most attractive aspect of performance-based regulation is its "third way" solution – avoiding the greater problems that come from either relying entirely on the market or relying on government experts to dictate in detail how businesses should act.

## Competing interests

The authors declare that they have no competing interests.

## Authors' contributions

SS and NS have both made substantial contributions to the conception and design of the project 2) have been involved in drafting the manuscript, and 3) have given final approval of the version to be published.
